# Magnetic Resonance Imaging in Cerebral Small Vessel Disease‐Related Depression: From Visual Scoring to Artificial Intelligence

**DOI:** 10.1002/cns.71188

**Published:** 2026-09-30

**Authors:** Chaofang Lei, Jiaxu Chen, Yilong Wang

**Affiliations:** ^1^ Department of Neurology, Beijing Tiantan Hospital Capital Medical University Beijing China; ^2^ China National Clinical Research Center for Neurological Diseases Beijing China; ^3^ School of Traditional Chinese Medicine Beijing University of Chinese Medicine Beijing China; ^4^ Guangzhou Key Laboratory of Formula‐Pattern of Traditional Chinese Medicine, School of Traditional Chinese Medicine Jinan University Guangzhou China; ^5^ Laboratory for Clinical Medicine Capital Medical University Beijing China; ^6^ National Center for Neurological Disorders Beijing China; ^7^ Advanced Innovation Center for Human Brain Protection Capital Medical University Beijing China; ^8^ Beijing Laboratory of Oral Health Capital Medical University Beijing China; ^9^ Beijing Municipal Key Laboratory of Clinical Epidemiology Beijing China; ^10^ Chinese Institute for Brain Research Beijing China

**Keywords:** artificial intelligence, cerebral small vessel disease, deep learning, depression, imaging biomarker, machine learning, magnetic resonance imaging, visual score

## Abstract

**Background:**

Cerebral small vessel disease (CSVD) is the key pathological basis of vascular depression. The precise identification of its neuroimaging markers is of core value for early diagnosis, elucidation of its pathological mechanism, and individualized treatment. Recent advances in magnetic resonance imaging (MRI) and artificial intelligence (AI) have enabled automated, high‐throughput characterization of CSVD‐related brain lesions. However, the translation of these technical advances into clinical tools for depression‐specific prediction and classification remains at an early stage.

**Results and Conclusion:**

This manuscript aims to summarize the application of traditional visual scoring systems in assessing the burden of CSVD and its association with depressive symptoms. Review the current status of imaging and AI research on CSVD‐related depression. To provide a direction for the development of more precise and efficient imaging diagnostic tools for the future, and ultimately promote the practical application and utilization of precision medicine in the field of CSVD‐related depression.

## Graphical abstract

Starting with MRI data acquisition, it presents the core steps and outputs of visual scoring, traditional machine learning, and deep learning along three evolving paths, and integrates them into the clinical application direction, illustrating the technological evolution from qualitative description to precise prediction.

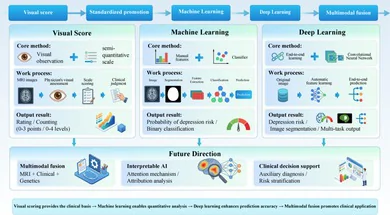

## 1 Introduction

Cerebral Small Vessel Disease (CSVD) refers to a group of pathological processes that affect the small arteries, arterioles, capillaries, and small veins of the brain. Its diagnosis is dependent on neuroimaging [1]. On magnetic resonance imaging (MRI), the typical imaging manifestations of CSVD include recent small subcortical infarcts (RSSI), presumed vascular‐origin lacunes, white matter hyperintensities (WMH), perivascular spaces (PVS), cerebral microbleeds (CMBs), and brain atrophy [2]. These imaging markers not only reflect pathological changes in the vessels but also often co‐occur with adverse neurological outcomes [3]. Increasing evidence suggests that CSVD is not only an important cause of cognitive impairment and stroke, but also a key pathological physiological basis for late‐onset depression, especially vascular depression [4].

The imaging assessment based on MRI provides a unique window for understanding the complex relationship between CSVD and depression. Traditionally, scales relying on expert visual scoring have been the standard for assessing the burden of CSVD and its associated clinical outcomes. In the field of depression, research has also found a positive correlation between CSVD total burden and depressive symptoms [5]. However, these visual scoring methods have inherent disadvantages, such as strong subjectivity, a time‐consuming nature, limited repeatability, and insensitivity to subtle changes [6]. Therefore, the development of more intelligent and adaptive segmentation tools has become an urgent need.

In recent years, with the rapid development of artificial intelligence, semi‐automatic and fully automatic image segmentation and quantification methods for imaging biomarkers of CSVD‐related depression have emerged. Especially with the introduction of machine learning and deep learning methods, new approaches have been opened up for automatically, objectively, and efficiently extracting subtle imaging biomarkers related to depressive symptoms from multimodal MRI data. Deep learning models can directly learn features from original images and automatically detect and segment various CSVD lesions [6]. This suggests that future research can draw on such data‐driven methods, integrating multimodal MRI features (such as DTI, SWI, and brain network connectivity properties), to construct imaging models that can more accurately identify and predict the risk of CSVD‐related depression. Through artificial intelligence, it is expected to discover imaging feature combinations specific to depression symptoms beyond traditional cognitive measures, thereby overcoming the subjectivity of visual scoring and the limitations of single indicators, and providing new possibilities for early identification, risk stratification, and individualized intervention.

## 2 Methods

This manuscript is a narrative review aimed at integrating the existing evidence on CSVD‐related depression in the imaging field. The selection of the review topic, the depth of the analysis, and the overall structure were determined through collaborative discussions among all authors.

The literature search covered multiple electronic databases, including CNKI, Wanfang, PubMed, Embase, and Web of Science. The search terms were tailored for each part of the review and included combinations of keywords related to CSVD, depression, MRI, visual scoring, AI, deep learning, and machine learning. There were no restrictions on the publication year. The included studies were based on their relevance to the research topic.

Research results were not subjected to formal quantitative synthesis but were integrated using narrative methods and organized into thematic sections that reflect key issues. Disagreements in research interpretation or relevance were resolved through consensus among the authors. The narrative design of the review aims to achieve a comprehensive and clinically meaningful synthesis of heterogeneous literature.

## 3 Interrelationships and Clinical Significance of Depression Related to CSVD

### 3.1 CSVD as the Pathological Basis of Vascular Depression

The vascular depression hypothesis emphasizes the central role of cerebrovascular diseases, especially CSVD, in the pathogenesis of mood disorders [7]. CSVD disrupts the structural and functional integrity of key neural circuits, particularly the frontal‐limbic‐subcortical network, leading to impairments in emotional regulation [8]. This circuit is a critical hub for emotional processing and executive functions, and vascular lesions related to CSVD can damage its white matter fiber connections, thereby impairing the efficiency of information transmission [9]. Studies have shown that patients with CSVD accompanied by depressive symptoms exhibit significantly reduced white matter network efficiency, including decreased global efficiency and increased characteristic path lengths, which directly reflects the structural damage to the frontal‐limbic circuit [10]. This reduction in network efficiency is associated with the severity of depressive symptoms, suggesting that the integrity of the white matter network is an important factor in maintaining normal emotional function [10]. Therefore, CSVD damages the structural framework of the emotional regulation circuit, constituting the core neuroanatomical mechanism of vascular depression.

The neurobiological basis of CSVD‐related depression involves the interaction of multiple pathological and physiological processes. Chronic hypoperfusion is an early and persistent feature of CSVD, causing chronic ischemic damage to deep white matter [11]. This hypoperfusion state is closely associated with dysfunction of the blood–brain barrier (BBB). The vascular pathology of CSVD, such as arteriosclerosis and amyloid angiopathy, damages the integrity of the BBB, leading to the leakage of plasma components, triggering neuroinflammatory responses and oxidative stress [12]. Neuroinflammation further exacerbates myelin loss and may directly damage neuronal and synaptic functions [13]. These processes work together to cause axonal damage and secondary disruptions in white matter network connections.

Additionally, endothelial dysfunction plays a key role in the pathogenesis of CSVD, and the related biomarker changes are also associated with the clinical manifestations of CSVD, possibly indirectly promoting the neurobiological process of depression by affecting cerebral blood flow autoregulation and BBB function [14]. Therefore, chronic hypoperfusion, BBB disruption, neuroinflammation, and endothelial dysfunction form a mutually reinforcing vicious cycle, jointly establishing the complex pathological and physiological basis of depression related to CSVD. Figure 1 illustrates the MRI imaging markers and mechanisms related to CSVD‐related depression.

**FIGURE 1:**
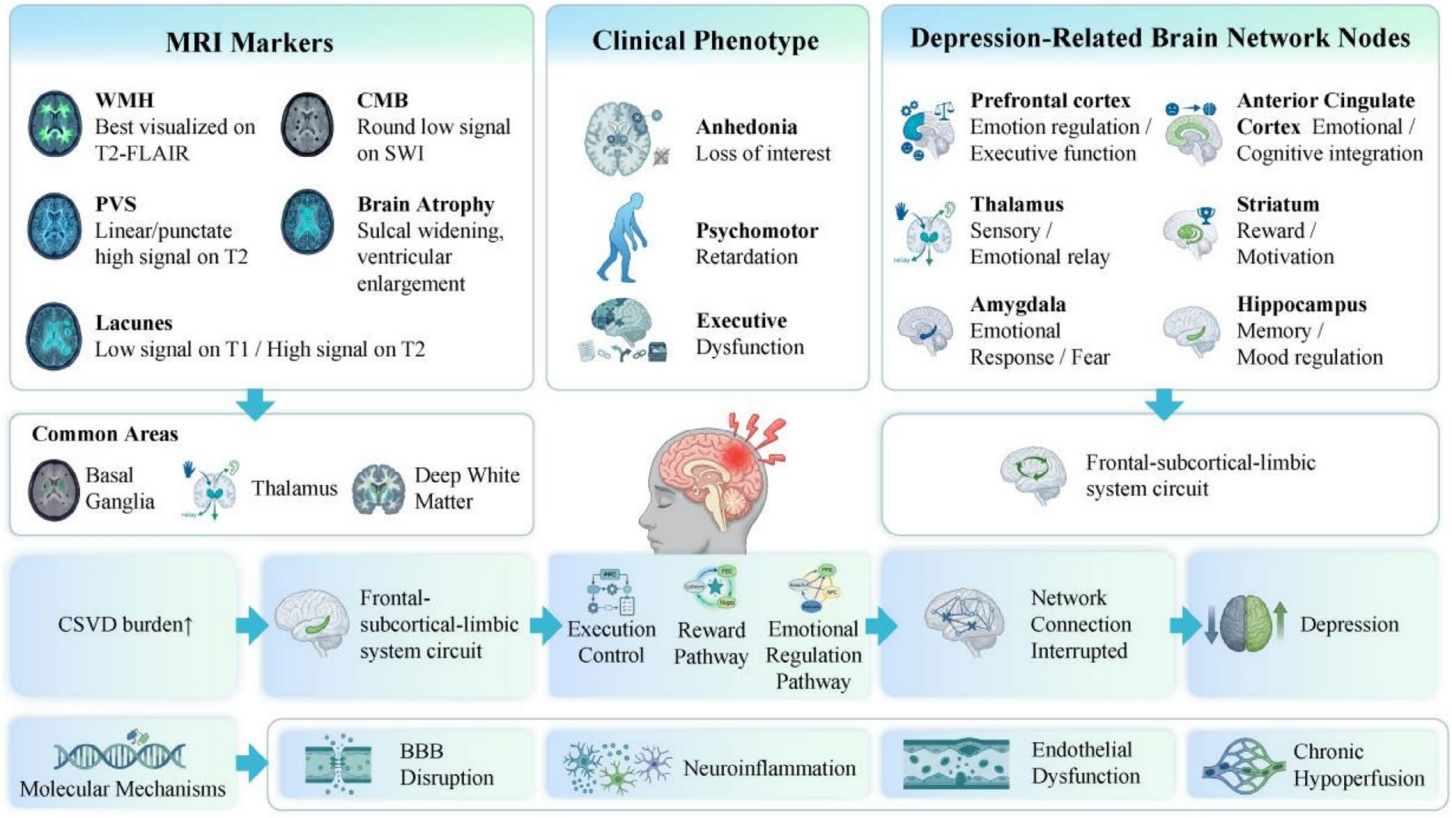
On the left, common MRI signs of CSVD (WMH, brain atrophy, CMBs, PVS, lacunes) are shown, mostly located in the basal ganglia, thalamus, and deep white matter. On the right, the clinical phenotypes of depression and key brain regions (prefrontal cortex, anterior cingulate cortex, amygdala, hippocampus, striatum, thalamus) are presented. At the bottom, the pathological mechanism is illustrated, depicting the entire process from imaging markers to brain network disruption and ultimately to depressive manifestations.

### 3.2 Clinical Requirements and Value of Image Biomarker Identification

The early identification and precise intervention of depression associated with cerebral small vessel disease are urgent needs in current clinical practice. CSVD, as a common, chronic, and progressive vascular disease, has common imaging manifestations in the elderly population [4, 15]. These imaging changes are not only indicators of vascular damage but are also significantly associated with an increased risk of depression. Studies have shown that the severity of CSVD is closely related to the diagnosis of depression, both before and after the occurrence of cerebral hemorrhage [16]. Therefore, identifying the early imaging biomarkers of CSVD through neuroimaging techniques such as MRI is crucial for screening out individuals at high risk of depression. For example, a study on survivors of intracerebral hemorrhage (ICH) found that the imaging burden related to cerebral amyloid angiopathy was significantly associated with the incidence of depression and resistance to antidepressant treatment [16]. This suggests that stratifying the assessment of CSVD subtypes (such as atherosclerotic vascular disease and cerebral amyloid angiopathy) and their severity based on imaging biomarkers, and predicting the short‐term outcome of ICH, can provide objective evidence for clinicians, enabling them to implement preventive psychological interventions for high‐risk individuals, strengthen the management of vascular risk factors, and formulate individualized follow‐up plans, ultimately achieving the goal of delaying the occurrence of depression or reducing its severity [17, 18, 19].

## 4 Application of Traditional Visual Rating Scales in the Assessment of Depression in CSVD

### 4.1 Common Visual Rating Scales 

The Fazekas scale is one of the most widely used semi‐quantitative assessment tools in studies of depression in cerebral small vessel disease. It is primarily used to evaluate the severity of white matter hyperintensities [20, 21]. This scale converts the complex imaging manifestations of white matter hyperintensities into a quantitative measure of overall white matter lesion burden [22]. Multiple studies have confirmed that the visual rating based on the Fazekas scale is significantly associated with the severity of depressive symptoms. For example, a cross‐sectional study on patients with cerebral small vessel disease found that the Fazekas score was positively correlated with the Hamilton Depression Scale score, indicating that the higher the visual score of white matter lesions, the more severe the depressive symptoms might be [23].

The modified Scheltens scale provides a more comprehensive assessment framework. Unlike the Fazekas scale, which mainly focuses on white matter lesions, it is primarily a regional WMH‐rating system. It is suitable for longitudinal studies of the relationship between WMH progression and clinical manifestations [24, 25]. Studies have shown that this comprehensive visual scoring system has a closer association with neuro‐psychiatric symptoms such as depression. For example, a study using the total burden score of cerebral small vessel disease (a comprehensive scoring method similar to the modified Scheltens scale) found that a higher total burden score was significantly associated with more severe depressive symptoms [26, 27].

For the assessment of lacunar cerebral infarction, the Standards for Reporting Vascular Changes on Neuroimaging (STRIVE‐2) provide a clear definition. According to STRIVE‐1, lacunes are circular or oval‐shaped lesions with a diameter of 3 to 15 mm, filled with liquid or cerebrospinal fluid‐like substance, typically located in the subcortical region [28, 29]. STRIVE‐2 no longer recommends relying solely on the diameter of the lesion to differentiate lacunes. Instead, it recommends making a comprehensive judgment based on the lesion morphology, the vascular space around the lesion, and the signal of the surrounding tissues [30, 31]. For the important hemorrhagic CSVD marker, the microbleed anatomic rating scale (MARS) and brain observer microbleed scale (BOMBS) provide specialized assessment frameworks [32, 33, 34].

The age‐related white matter change (ARWMC) score is another systematic visual scoring tool that evaluates the distribution and severity of white matter lesions across different brain regions (e.g., the frontal and parietal lobes) [35, 36]. This regional assessment method helps to reveal the potential links between the spatial distribution patterns of white matter lesions and specific clinical symptoms (such as depression), and the ARWMC score may be related to depression [37, 38]. The core value of these visual scoring scales lies in converting qualitative, complex MRI manifestations into repeatable, comparable quantitative scores or grades [22, 39]. The comparison of common visual scoring scales related to cerebral small vessel disease is presented in Table 1.

**TABLE 1:** Comparison of visual rating scales for CSVD.

Scale	Target lesions	Scoring dimensions/content	Advantages	Disadvantages	Applicable scenarios
Fazekas scale	Periventricular white matter Deep white matter	White matter hyperintensities in the periventricular region (rated on a scale of 0 to 3) and those in the deep white matter (rated on a scale of 0 to 3) were each semi‐quantitatively classified, with a total score ranging from 0 to 6	Simple and efficient, with strong clinical operability Widely applied, with abundant accumulated research data Its correlation with the severity of depression has been extensively verified	This scale is insensitive to detailed changes in WMH and is subjective, with blurred boundaries between different grading levels	Large‐scale epidemiological screening Rapid clinical assessment of overall WMH burden Preliminary screening tool for depression risk
Modified Scheltens scale	Periventricular white matter Deep white matter Basal ganglia region (including putamen, caudate nucleus, globus pallidus) Brainstem, cerebellum	Periventricular white matter hyperintensities were scored from 0 to 6 points, and deep white matter hyperintensities were scored from 0 to 24 points	It covers the entire brain area, includes white matter lesions and deep gray matter damage, and is more closely associated with neurological and psychiatric symptoms (including depression)	The scoring rules are complex, the procedure is time‐consuming, and the inter‐rater consistency is slightly lower	Clinical research at the intersection of neurology and psychiatry
MARS	Cortex, subcortical white matter, basal ganglia region, thalamus, cerebellum, brainstem	Classification was based on the total number of microbleeds counted using the MARS scale	Detailed classification of microbleeds in the brain lobes	No distinction was made based on microbleed size	Research on the relationship between the distribution pattern of CMBs and depression
BOMBS	Grouped by anatomical regions (cerebral lobes, deep structures, cerebellum, brainstem)	A semi‐quantitative assessment of the regional distribution and number of CMBs was performed	It is easier to operate than MARS and more suitable for routine clinical applications	Relies on visual identification, with sensitivity influenced by observer experience Has limited ability to distinguish microbleeds from confounding signals (e.g., calcification and iron deposition)	Rapid assessment of CMBs burden in large‐scale clinical studies Simplified alternative to MARS for routine clinical records
ARWMC	Frontal area, parieto‐occipital area, temporal area, inferior brain area and basal ganglia area	The severity of WMH in the left and right brain regions was classified on a scale of 0 to 3, and the total score (ranging from 0 to 30) was calculated	Reveals the spatial distribution of lesion sites	Regional division criteria vary considerably between studies	Association between WMH spatial distribution and depressive symptom dimensions Relationship between frontal‐limbic circuit damage and emotional dysregulation

### 4.2 Evidence of the Association Between Visual Scores of CSVD and Depressive Symptoms

A study found that the overall volume of WMH was significantly associated with an increased risk of depression (OR = 1.07 [95% CI 1.05–1.10]) [40]. This finding is consistent with the research conclusion on late‐life depression (LLD), that is, WMH is the most common imaging finding associated with CSVD and LLD, and its volume increase is related to the aggravation of depressive symptoms [41]. The basal ganglia and thalamus are key brain regions that regulate emotions and motivation, and are also important components of the frontal‐limbic circuit. The lacunar infarctions in these regions may disrupt the neural circuits connecting the frontal cortex and the limbic system, leading to dysfunction in emotional regulation and thereby increasing the risk of depression [42].

CMBs is a microvascular hemorrhagic marker of CSVD, and its distribution pattern is associated with specific dimensions of depressive symptoms. Increasing evidence suggests that microbleeds located in the frontal lobe, basal ganglia region, and thalamus may be related to certain core symptoms of depression, such as lack of motivation. Lack of motivation is a common feature of depression and apathy, and apathy is particularly common in CSVD patients. A cross‐sectional study of elderly patients with CSVD in China found that apathy was positively correlated with the total CSVD load score [43]. However, the distinction between depression and apathy is particularly pertinent in CSVD. Apathy—characterized by diminished motivation, reduced initiative, and emotional indifference—is prevalent in CSVD patients due to disruption of frontal‐striatal‐pallidal‐thalamocortical circuits. Although apathy frequently co‐occurs with depression, it is clinically and neurobiologically distinct. Notably, apathy correlates more strongly with executive dysfunction and white matter tract disruption in the frontal cortex regions, whereas depression is more closely associated with limbic circuitry [44, 45]. This distinction has profound implications for AI‐based models: a model trained to classify “depression” using HAMD scores may inadvertently learn features related to apathy or cognitive slowing, rather than core mood disturbance, if the training labels do not parse these dimensions. To date, no deep learning study has attempted to disentangle depression from apathy in CSVD populations using multimodal MRI, representing a critical direction for future research.

Brain atrophy, especially global brain or hippocampal atrophy, as the terminal manifestation of CSVD, also has visual assessment indicators that are associated with depressive symptoms. Clinical studies have observed that as the overall burden of CSVD increases, the prevalence of brain atrophy also rises, and it is positively correlated with the depression score [46]. However, this association should be interpreted with caution. In the elderly population, brain atrophy, especially hippocampal atrophy, is often the result of the combined effects of neurodegenerative pathology and vascular pathology [47]. This suggests that in clinical practice, when encountering elderly patients with predominant manifestations of brain atrophy and depression, we need to consider the cerebral small vessel disease burden and other possible neurodegenerative disease markers comprehensively to better understand the pathological mechanism and formulate individualized treatment plans.

## 5 From Visual Scoring to Automatic Quantitative Analysis: Radiomics and Machine Learning

### 5.1 Fully Automatic or Semi‐Automatic Segmentation and Quantification of CSVD Lesions

With the widespread adoption of MRI technology, imaging studies of CSVD have shifted from traditional qualitative visual assessment to computer‐assisted quantitative analysis. To achieve more accurate and reproducible lesion quantification, researchers have developed various fully automatic or semi‐automatic image processing algorithms and software tools. These methods mainly rely on traditional image processing techniques such as threshold segmentation, region growing, and atlas registration, and can efficiently identify and segment WMH from MRI [48]. For example, software tools such as kNN‐TTP, BIANCA, and UBO Detector have been widely used in clinical research. They can automatically calculate key quantitative indicators, such as total volume, lesion count, and the spatial distribution of WMH, thereby significantly improving research efficiency and reducing inter‐observer differences [49, 50]. These automatic segmentation tools provide a technical foundation for large‐scale cohort studies and longitudinal follow‐ups, enabling detailed assessment of CSVD burden. However, it is worth noting that automated segmentation of WMH, CMBs, PVS, or lacunes quantifies CSVD lesions; it does not, by itself, identify a depression biomarker. Previous studies have shown that imaging markers are associated with the risk of depression. Whether the segmented volume/quantity indicators can serve as specific biomarkers for depression still requires a comprehensive judgment based on functional imaging, cortical morphology, and clinical information.

### 5.2 Machine Learning‐Based Radiomics

Radiomics, an emerging quantitative analysis method, extracts numerous quantitative features from MRI images that are difficult for the human eye to identify, in a high‐throughput manner, providing a powerful tool for the precise identification of CSVD‐related depression [40, 51]. These features include not only traditional intensity features but also complex features extracted by advanced algorithms, such as texture and intensity, which can comprehensively depict the microstructural heterogeneity of brain tissue [52]. For example, in the core imaging biomarker of WMH, radiomics can quantify its internal heterogeneity, which may reflect the pathophysiological processes underlying white matter damage and be related to the emergence of depressive symptoms [53]. Compared with traditional methods that rely solely on volume or visual scoring, radiomics can reveal more subtle and complex imaging pattern differences, thereby transcending the limitations of human visual assessment and opening new avenues for understanding the neurobiological basis of CSVD with depression [54].

## 6 Substantial Advancement of Deep Learning in CSVD Image Analysis

### 6.1 Application of CNN in Automatic Segmentation and Quantification of CSVD Lesions

Convolutional neural networks (CNNs), as the mainstream deep learning architecture for processing medical images, have a core advantage: they automatically learn abstract features from low‐level to high‐level in the original image data, thereby avoiding the cumbersome, subjective manual feature extraction process in traditional methods [55, 56]. CNNs have shown great potential for the automatic segmentation and quantification of CSVD imaging markers, with the advantage of enabling precise, efficient, and repeatable lesion identification. The CNN architecture, U‐Net, has been successfully applied to the automatic segmentation of key CSVD markers, such as WMH [55]. Automatic quantification features based on CNNs have been shown to reveal new correlations with depressive symptoms, surpassing traditional visual scoring.

### 6.2 Deep Learning Model for Depression Assessment in CSVD

An artificial intelligence model provides a powerful technical framework for directly identifying the depressive state associated with CSVD from multimodal MRI images. These models directly use either raw or preprocessed T1‐weighted images (T1WI), DTI, etc., as inputs, thereby providing an efficient, automated tool for subsequent depression‐related analysis [26, 57, 58, 59]. These models can automatically learn optimal feature combinations relevant to the task and may discover previously unknown and complex cross‐modal interaction patterns, thereby achieving higher classification performance. For example, a study used graph theory analysis combined with DTI data to find that CSVD patients with depressive symptoms had reduced efficiency of the white matter structure network, and abnormal network connections were related to the severity of depression [58]. This suggests that deep learning models may capture these complex network features reflecting neural circuit dysfunction. Although research on directly applying models for CSVD depression classification is still in its early stages, the related work provides insights into future directions.

## 7 Quantitative Imaging and AI Methods

### 7.1 Advanced Diffusion MRI Techniques

Peak width of skeletonized mean diffusivity (PSMD) is a comprehensive indicator reflecting the heterogeneity of white matter microstructure and is sensitive to diffuse vascular damage. It overcomes the dependence of traditional DTI indicators on lesion‐area selection [54]. A study found that in patients with late‐onset depression (LLD), the negative correlation between PSMD and cognitive processing speed was significantly stronger than that in healthy controls, and PSMD outperformed traditional SVD markers in predicting executive function [54]. This suggests that PSMD may be a sensitive marker for capturing cognitive deficits related to depression in the context of CSVD. Currently, there are no studies directly examining the association between PSMD and the severity of depressive symptoms (such as HAMD score) in CSVD. Traditional DTI parameters (FA, MD) are affected by vascular edema and increased extracellular free water, whereas free water imaging can quantify fiber tract damage (tissue fractional anisotropy, tFA) and extracellular fluid increase (free water fraction, FW) [53]. This distinction is particularly crucial in CSVD, as the disruption of the blood–brain barrier and vascular leakage are the core pathological processes. A study found that in CSVD patients, the HAMD depression score was significantly correlated with the free water fraction (FW). This pattern is opposite to that of healthy elderly populations, suggesting that the neural mechanism of CSVD depression may be dominated by vascular leakage and inflammation rather than simple white matter fiber damage [53]. This finding has important clinical significance. If verified in CSVD depression patients, free water imaging may become a biomarker for monitoring the efficacy of anti‐inflammatory or vascular protection treatments. Neurite orientation dispersion and density imaging (NODDI) distinguishes intracellular, extracellular, and cerebrospinal fluid signals using a multi‐compartment model, yielding parameters such as the neurite density index (NDI) and orientation dispersion index (ODI), which are more specific to microstructural pathology than traditional DTI [60]. Currently, the application of NODDI in CSVD is mainly focused on cognitive impairment. A study found that the ODI and free water fraction (FISO) indices of NODDI can sensitively monitor the microstructural changes in the hippocampal subregion of CSVD patients. Still, no study has yet associated it with depressive symptoms [60]. Therefore, the application of NODDI in CSVD depression is in an exploratory direction.

### 7.2 Advanced Segmentation and Transformer Architectures

As an adaptive framework, nnU‐Net can automatically optimize preprocessing, network architecture, and post‐processing strategies and performs exceptionally well in various multi‐class medical segmentation tasks. The precise quantification of WMH is the basis for CSVD image assessment, but traditional segmentation tools suffer from bias and poor reproducibility [61]. A systematic validation study showed that nnU‐Net performed well in WMH segmentation and reduced the sample size required for detecting treatment effects by 41% compared to the benchmark algorithm [61]. However, there are no studies yet that apply nnU‐Net or similar advanced segmentation tools to the clinical correlation analysis of CSVD‐related depression images. Current quantitative studies on CSVD‐depression still mainly rely on traditional manual or semi‐automatic segmentation methods, and this technological gap constitutes a clear void. The transformer architecture can capture complex interaction patterns between distant brain regions through self‐attention mechanisms and is theoretically well‐suited to modeling diffuse and multifocal lesions such as CSVD [2, 62]. Preliminary studies have explored the application of transformer architecture in the analysis of multimodal CSVD images (such as the CSVDtransformer model), but its goals are mostly for predicting CSVD load scores rather than depression status [62]. The application of a transformer in CSVD depression classification is currently unexplored. Considering that CSVD depression is believed to involve extensive functional disorders in the frontal‐limbic‐subcortical circuit, the ability of Transformer to capture cross‐brain region interactions has theoretical appeal, but it requires empirical validation.

## 8 Strategies for the Integration of Multimodal MRI and Deep Learning

### 8.1 Joint Analysis of Structural MRI and Diffusion Tensor Imaging

The joint analysis of structural magnetic resonance imaging (sMRI) and diffusion tensor imaging represents an important advance in the research on imaging markers for CSVD, moving from macroscopic morphology to the integration of microscopic structure. The DTI technique measures the diffusion properties of water molecules within white matter fiber tracts, enabling quantitative assessment of the microstructural integrity of normal white matter (NAWM) in conventional MRI. It has significant advantages for detecting myelination and developmental changes in the brain's white matter microstructure [63, 64]. By combining T2‐fluid‐attenuated inversion recovery (FLAIR)/T1 images reflecting macroscopic lesions with DTI data reflecting the microstructural integrity of white matter, a complete pathological map from macroscopic lesions to microscopic network damage can be constructed. For example, studies have shown that even when the volume of WMH is similar, patients with CSVD and depressive symptoms have significantly lower white matter network efficiency (such as global efficiency) than those without depression, suggesting that microstructural damage may play a more critical role in the occurrence of emotional disorders [10]. This multimodal fusion strategy has been proven to be able to more comprehensively assess neural circuit damage and provide a new perspective for understanding the neural basis of CSVD‐related depression [58].

### 8.2 Integration of Functional MRI and Structural Information

Combining functional connectivity information from resting‐state functional magnetic resonance imaging (rs‐fMRI) with structural damage information from CSVD to construct a “structural‐functional” coupling model is a key path toward an in‐depth understanding of the neural mechanisms underlying depression in CSVD. The rs‐fMRI study revealed abnormal functional connectivity within emotion regulation‐related networks in CSVD patients. A study on the emotional dysregulation in CSVD patients found that depression was significantly correlated with the weakened functional connectivity between the salience network and the language network, as well as within the language network [65]. Therefore, integrating the structural damage (such as the microscopic structural changes in specific white matter pathways) with the resulting functional network connection disarray enables a more systematic elucidation of how CSVD, by disrupting the information transmission efficiency of brain networks, ultimately leads to the generation of depressive emotions from the perspective of “structural damage—functional compensation/abnormality” [58]. This integrated model surpasses the limitations of a single modality and provides a powerful framework for revealing the complex network pathophysiology of depression related to CSVD. A summary of automated quantitative and artificial intelligence studies in CSVD neuroimaging is shown in Table 2.

**TABLE 2:** Summary of automated quantitative and AI studies in CSVD neuroimaging.

Study	Cohort size (*n*)	MRI modalities	Algorithm/architecture	Target task	Performance metrics	Validation strategy	Depression as endpoint?
Zhao et al. [66]	163 (105 internal + 58 external)	Multisequence MRI (T1WI, T2WI, FLAIR, and SWI)	Deep learning‐based automated segmentation	WMH, CMBs, lacunes, and enlarged perivascular spaces (EPVSs)	Dice: WMH = 0.85, CMBs = 0.74, lacunes = 0.76, EPVS = 0.75; Pearson *r* = 0.947–0.978	Internal + external validation	No
Gibson et al. [67]	> 700 (7 multi‐site cohorts)	FLAIR	CNN (segcsvdWMH, hierarchical segmentation)	WMH segmentation	Mean dice = 0.86 ± 0.08; mean volume *r* = 0.99 ± 0.01	Multi‐site heterogeneous test datasets	No
Lin et al. [57]	198 (training *n* = 113; external validation 1 *n* = 85, externalvalidation 2 *n* = 102)	Structural MRI, fMRI, and DTI	XGBoost (9 machine learning classifiers compared)	CSVD patients with depression, CSVD patients without depression	AUC = 0.958 (training), 0.893 (external validation 1), 0.917 (external validation 2); accuracies = 0.929, 0.871, 0.853	Multi‐center external validation (2 external cohorts)	Yes
Gu et al. [58]	36 CSVD patients with depressive state (CSVD+D), 34 CSVD patients without depressive state (CSVD−D), and 45 controls	DTI	TBSS + graph theory (structural network analysis)	White matter microstructure–depression association	FA/MD changes; structural network metrics correlated with HAMD	Not applicable (observational)	Yes
Lan et al. [59]	CSVD+D, *n* = 24; CSVD−D; *n* = 35	DTI + serum proteomics	Correlation analysis + protein module identification	DTI diffusivity–inflammation–depression association	Two protein modules associated with DTI diffusivity in CSVD+D	Not applicable (observational)	Yes
Zhang et al. [26]	22 CSVD patients with depression (CSVD+D) and 34 healthy controls (HC)	T1WI	Individualized differential structural covariance network (IDSCN)	CSVD+D vs. HC classification	Differential edges correlated with HAMD scores	Not applicable (observational)	Yes

## 9 Current Challenges and Solutions for Artificial Intelligence

### 9.1 Data Requirements and Annotation Bottlenecks

Artificial intelligence has shown great potential for identifying imaging biomarkers of depression associated with cerebral small vessel disease; however, its successful application depends heavily on large‐scale, high‐quality, and accurately labeled training data [68, 69]. However, acquiring medical imaging data poses multiple challenges. Firstly, obtaining high‐resolution MRI data that meets ethical requirements and contains detailed clinical information is costly and complex [70, 71]. Secondly, generating reliable labels for this data is another key bottleneck. For example, the diagnosis of depression requires assessment based on standardized neuropsychiatric scales, while the precise delineation of CSVD lesions (such as lacunes, white matter hyperintensities) requires manual completion by experienced neuroimaging experts, both of which are extremely scarce and time‐consuming [59]. This data scarcity directly limits the generalization ability of deep learning models, making it difficult for them to be stably applied in a wider range of clinical populations.

To address the challenges of data scarcity and labeling difficulties, researchers have proposed a variety of innovative solutions. Transfer learning is an effective strategy, where a pre‐trained model on a large natural image dataset (such as ImageNet) is utilized to transfer the general feature extraction capabilities learned by the model to the field of medical imaging, and then relatively small amounts of MRI data of CSVD are used for adjustment [72, 73]. This method can significantly reduce the demand for large‐scale labeled data in specific fields. Additionally, constructing multi‐center, large‐scale shared datasets is the fundamental way to solve the data bottleneck. Through international cooperation, integrating imaging and clinical data from different medical institutions can greatly expand the sample size and diversity [40]. These methods combined provide a feasible technical path for developing high‐performance brain small vessel disease depression image recognition models with limited labeled resources.

### 9.2 Interpretability and Clinical Credibility of the Model

Deep learning models have shown great potential in identifying imaging markers for CSVD‐related depression. However, their inherent “black box” nature poses a major obstacle for clinical application. These models, especially complex CNNs, can learn from high‐dimensional imaging data and make accurate predictions. Still, their internal decision‐making logic and the specific imaging features they rely on are often difficult for humans to understand [73]. This opacity makes it challenging for clinicians to trust the model's output, hindering its integration and acceptance as an auxiliary diagnostic tool in routine clinical practice. Therefore, improving the model's interpretability and making its decision‐making process transparent is a crucial step toward promoting the transition of related artificial intelligence technologies from research to clinical practice and enhancing their clinical credibility.

To uncover the “black box” of deep learning models, researchers are actively developing methods to interpret artificial intelligence. The attention mechanism is an important technique that can visualize the areas of the model's “focus” when making decisions. By generating heat maps, it is possible to visually display which brain regions or image features contribute the most to the model's predictions. For example, when using deep learning to analyze DTI data of CSVD to identify depression‐related microstructural changes, the attention mechanism may reveal that the model focused on the white matter fiber integrity of the frontal lobe or the limbic system, which is consistent with the known depression neural circuit theory, thereby enhancing the biological interpretability of the results [74, 75]. Moreover, when building machine learning models based on routine clinical data for CSVD risk stratification, using interpretability frameworks such as SHapley Additive exPlanations (SHAP), it is possible to clearly show the contribution of each feature (such as age, uric acid, blood pressure, etc.) to the model's predictions, thereby enhancing the transparency of the model and the trust of clinicians [76].

However, interpretability should be treated with caution. These visual results should not be automatically equated with biological validity. As pointed out in the literature, these graphs reflect the computational attention of the model. They may capture interfering variables (such as skull artifacts, motion, or scanner‐specific noise), rather than true neurobiological signals [77]. To rigorously validate the claim of interpretability, independent biological confirmation (such as consistency with known lesion defect maps or autopsy histological results) is required. However, to date, no such verification has been conducted in any artificial intelligence research on CSVD‐related depression. Therefore, although we propose that these visualization techniques have promise, we emphasize that they still belong to generating hypotheses rather than confirming hypotheses.

### 9.3 Generalization Ability and Standardization

In research on identifying MRI‐based CSVD‐related depression imaging markers, the generalizability and standardization of AI models are key challenges that determine whether they can be successfully applied in real clinical settings. Model performance degradation across different scanning devices, sequence parameters, and populations is a common problem. For instance, the imaging manifestations of CSVD, such as WMH, lacunes, CMBs, and PVS, are dependent on the MRI acquisition protocol [78, 79]. Scanning devices from different manufacturers, field strengths (e.g., 1.5T and 3.0T), and different sequence parameters (such as TR, TE, slice thickness, and resolution) all lead to significant differences in image contrast, signal‐to‐noise ratio, and artifacts, thereby affecting the model's ability to identify and segment lesions [80, 81, 82]. Moreover, the heterogeneity of the study population, such as different races, ages, comorbidity status (such as hypertension, Alzheimer's disease), and different subtypes of CSVD, can cause the distribution of imaging features to shift, making a model trained on one cohort perform poorly on another [47, 83, 84, 85, 86]. Therefore, improving the model's generalization ability is a prerequisite for its clinical translation.

To address these challenges, a series of technical measures must be adopted to enhance the model's robustness and universality. Firstly, data preprocessing standardization is a fundamental and crucial step. This involves uniform operations, such as intensity normalization, bias field correction, and spatial standardization, applied to the original images to reduce the impact of non‐biological variations [87, 88]. Secondly, domain adaptation techniques are an effective strategy to improve the model's performance across different centers and devices. These methods aim to transfer knowledge from the source domain to the target domain, even when the data distributions differ [89]. For example, through adversarial training or feature alignment, the model learns domain‐invariant features that are insensitive to imaging differences [90, 91]. Additionally, designing networks that are robust to imaging differences is also crucial. For instance, using an attention mechanism enables the model to focus more on regions of pathological significance rather than background noise, and leveraging multi‐scale feature fusion enhances the model's adaptability to images of different resolutions [92]. Finally, constructing a large‐scale, multi‐center, and standardized CSVD imaging database is the fundamental solution. By collecting standardized data across devices and populations, a more representative model can be trained, and a unified standard for imaging biomarker evaluation can be established.

### 9.4 Methodological Quality and Reporting Standards

Beyond summarizing the technical approaches and reported performance metrics, a critical appraisal of methodological quality is essential for interpreting the current evidence base. The AI in medical imaging community has established two complementary reporting guidelines: the Checklist for Artificial Intelligence in Medical Imaging (CLAIM), which comprises 42 items covering study design, data handling, model development, and evaluation specific to imaging AI, and the TRIPOD+AI statement, a 27‐item checklist for reporting prediction model studies regardless of whether regression or machine learning methods are used [93, 94]. In the following, we apply key items from these frameworks to critically evaluate the studies summarized.

Overfitting refers to the situation where the model captures noise rather than the actual signal. This remains a common risk in small‐sample neuroimaging studies [77]. Among the CSVD‐related depression studies reviewed, few report explicit strategies to mitigate overfitting beyond basic train‐test splits. A particularly insidious source of data leakage occurs when images or image slices from the same patient are distributed across training and test sets. This practice inflates performance estimates because the model effectively learns patient‐specific features rather than generalizable disease patterns. In CSVD research, where longitudinal or multi‐slice acquisitions are common, ensuring participant‐level (rather than slice‐ or image‐level) data splitting is mandatory. The CLAIM 2024 explicitly requires reporting of how data were partitioned and whether any individuals were represented in multiple partitions [95]. Beyond improper splitting, data leakage can occur through preprocessing steps (e.g., normalization using statistics computed from the entire dataset before splitting), feature selection performed on the full cohort, or—in longitudinal settings—using future information to predict past outcomes (temporal leakage). None of the CSVD‐related depression AI studies we identified explicitly reported guarding against these leakage pathways.

CSVD with clinically significant depression represents a minority subgroup in most cohorts, leading to class imbalance that biases models toward the majority class. Standard mitigation strategies include oversampling (e.g., SMOTE), undersampling, or class‐weighted loss functions [77]. However, these techniques introduce their own complexities. For instance, synthetic oversampling in high‐dimensional imaging feature spaces may generate unrealistic samples.

Multi‐center studies are essential for generalizability but introduce variability from different MRI scanners, field strengths, acquisition protocols, and site‐specific preprocessing pipelines. This heterogeneity can overwhelm the biological signal if not properly modeled. The use of harmonization techniques (e.g., ComBat for neuroimaging features) is increasingly recommended. Still, it remains rare in CSVD‐depression studies, particularly those employing deep learning on raw images, where the model architecture may implicitly learn site effects.

External validation involves testing the developed model on data from an independent institution or dataset. This is the standard for evaluating the general applicability of a model [94]. However, as shown in Table 2, the majority of CSVD‐related depression AI studies rely on internal validation (train‐test splits or cross‐validation within a single cohort). Only the study by Lin et al. [57] included external validation cohorts, and even in that case, the sample sizes were modest. The absence of robust external validation limits the clinical translatability of current models.

Although most studies have reported discriminatory indicators (such as accuracy rate, AUC), few have reported on the calibration situation, that is, the consistency between the predicted probability and the actual outcome. A model can have high AUC but poor calibration (e.g., consistently overestimating or underestimating risk), making it unsafe for clinical decision‐making. TRIPOD+AI explicitly requires reporting of calibration measures (e.g., calibration plots, Brier scores) [94, 96]. Among the CSVD‐related depression studies reviewed, none reported calibration statistics, representing a significant gap.

Finally, statistical performance (AUC, accuracy) does not equate to clinical utility. The TRIPOD+AI framework emphasizes that prediction models should be evaluated in terms of their intended clinical decisions and net benefit [94]. For CSVD‐related depression, a clinically valuable model is needed to demonstrate that its predictive results can significantly alter management decisions (such as the initiation of antidepressants, referral to a specialist) or improve patient prognosis. However, such evidence is currently not clear in the existing literature.

## 10 Future Outlook and Research Directions

### 10.1 Integrating Multimodal and Multiscale Information

Future research should focus on developing more effective strategies for integrating multimodal and multiscale information to construct a more comprehensive and robust CSVD depression prediction and diagnosis model. This requires going beyond traditional structural MRI and integrating structural, diffusion, functional, and emerging MRI sequence information more effectively. For example, in addition to the conventional T1 and T2‐FLAIR sequences, DTI can reveal changes in white matter fiber integrity and structural network topological properties [58], while rs‐fMRI can assess functional connectivity abnormalities in emotion‐processing‐related networks (such as the salience network and language network) [65]. Emerging techniques, such as quantitative susceptibility mapping, may provide information on iron deposition [97]. At the same time, non‐imaging data, such as genetic information, fluid biomarkers, and detailed clinical scale scores, are crucial for understanding the disease mechanism and improving model performance.

### 10.2 Personalized Medicine and Precision Psychiatry

The precision medicine and personalized treatment for depression related to CSVD are the core directions for future development. The ultimate goal is to build an intelligent system that integrates multi‐dimensional data. This system will combine multi‐source data, such as neuroimaging, genetic information, clinical phenotypes, and environmental factors, and use AI for in‐depth mining and modeling to achieve personalized prediction of depression risk, classification of pathological mechanisms, and treatment decision support for CSVD individuals. Promoting the classification of CSVD depression subtypes based on imaging markers is a key step in achieving precision psychiatry practice. Neuroimaging, especially multimodal MRI, can non‐invasively reveal the structural and functional changes in the brain of CSVD patients, and these changes have specific associations with depressive symptoms [98, 99]. However, CSVD includes various heterogeneous imaging manifestations and may cause depression through different mechanisms [100]. Using machine learning methods to cluster analyze multiple imaging markers of CSVD depression patients has initially identified subtypes with different imaging feature combinations and clinical prognoses [57]. This suggests that we can further explore the correspondence between these imaging subtypes and depressive phenotypes. For example, the study of brain white matter network disorders in patients with CSVD and microbleeds shows that the efficiency of nodes in the default mode network (DMN) regions (precentral gyrus, middle cingulate gyrus, middle temporal gyrus) and the left insula is significantly negatively correlated with the depression score [101]. Through this fine‐grained subtype classification, clinicians can go beyond the “one‐size‐fits‐all” treatment model and select more targeted therapies for different patient subtypes. For example, in the subtype with active inflammation, anti‐inflammatory treatment can be considered, and in the subtype with blood–brain barrier leakage, drugs to stabilize endothelial function can be explored, thus truly moving toward individualized, precision psychiatry interventions.

### 10.3 Clinical Heterogeneity and Construct Validity

A critical limitation of the current literature, which is the substantial clinical heterogeneity in defining and measuring “depression” across studies. The majority of visual rating and MRI‐quantitative studies employ dimensional symptom scales (e.g., HAMD, GDS‐15, CES‐D, PHQ‐9) rather than structured clinical interviews for DSM/ICD‐based MDD diagnosis. While pragmatic for large cohort studies, this approach conflates clinically significant depression with subsyndromal symptoms, which may represent different neurobiological substrates. Meanwhile, the generic term “depression” into its relevant clinical subtypes in the CSVD context: (a) Vascular depression is mainly observed in patients with stroke and cerebral small vessel diseases, and is characterized by high signal intensity in the white matter on imaging [102]; (b) post‐stroke depression (PSD)—depression occurring after a clinically overt cerebrovascular event, which may involve distinct inflammatory and lesion‐location mechanisms [103, 104]; (c) late‐onset depression (LOD)—first‐episode depression occurring in late life, often with underlying CSVD pathology but without a clear stroke history [105]; (d) depression in MRI‐defined CSVD—It may be related to the progression of clinical depressive symptoms and the severity of brain injury [106]. These subtypes have overlapping but non‐identical pathophysiological mechanisms, and the current literature—whether using visual scales or AI—rarely stratifies analyses by these categories, limiting generalizability.

Furthermore, we emphasize the critical distinction between cross‐sectional associations and longitudinal predictive relationships. The majority of CSVD‐depression studies summarized in Table 2 are cross‐sectional. These findings, while valuable for generating hypotheses, cannot establish causality or predict future depression risk. Only a handful of cohort studies have examined whether baseline CSVD burden predicts incident depression over time, and even fewer have applied machine learning to this longitudinal prediction task. We think against overinterpreting cross‐sectional associations as evidence of predictive utility, and we identify the longitudinal prognostic AI models as a research direction in the field.

### 10.4 Emerging Techniques Warranting Future Investigation

Quantitative Susceptibility Mapping (QSM) mainly measures the brain iron deposition related to neurodegeneration in the basal ganglia. In CSVD, QSM‐detected iron burden in the caudate and putamen correlated with cognitive and motor dysfunction [107]. However, its association with depressive symptoms in CSVD remains unexplored (evidence level: established in cognitive/motor domains, exploratory for depression). DTI‐ALPS (Diffusion Tensor Image Analysis Along the Perivascular Space) assesses the function of the cerebrospinal fluid system by measuring the diffusion coefficient of water in the perivascular spaces. Preliminary studies have shown that the ALPS index is associated with executive function deficits in CSVD and gait disorders caused by white matter damage [108]. Currently, no studies are exploring the relationship between DTI‐ALPS and depressive symptoms in patients with CSVD (evidence level: highly preliminary). Federated learning enables multi‐institutional model training without sharing the original patient data. This is particularly important for CSVD depression, as there is a lack of large‐scale datasets with clear phenotypic characteristics. To date, no federated learning research has been applied to the classification of CSVD depression (evidence level: conceptual/technical proposal). The foundation model (such as a large‐scale pre‐trained visual transformer) has the potential for zero‐shot or few‐shot adaptation in the CSVD depression classification task. Currently, there is no published relevant evidence in this specific field (evidence level: speculative, needs to be proven through experiments).

## 11 Conclusions

The research on imaging biomarkers of depression‐related CSVD has gradually advanced from macroscopic morphological assessment to the microscopic structural and functional network levels. It is moving toward a new stage of AI‐driven, precise identification. This evolutionary process not only reflects the rapid progress of neuroimaging technology but also deepens understanding of the disease's pathophysiological mechanisms.

Unlike previous reviews that focused on the general application of artificial intelligence in CSVD or specific patterns (such as lesion segmentation), this review has distinct characteristics. We pointed out that the clinical heterogeneity of the definition of depression is an important challenge in the development of artificial intelligence. At the same time, we used the established reporting framework (CLAIM, TRIPOD+AI) to assess the methodological rigor, revealing common flaws in model validation. Meanwhile, apathy, depressive symptoms, DSM/ICD‐defined major depression, late‐life depression, and post‐stroke depression represent distinct constructs with different clinical implications and should not be conflated. Therefore, we believe that depression is a priority but an understudied target. This review provides a roadmap for future research, aiming to bridge the gap between the technical development of artificial intelligence and the clinically significant prediction of depression in CSVD.

However, we also acknowledge that the evidence base for artificial intelligence in the field of CSVD‐related depression is limited. This gap is precisely the main motivation for our current review. We aim to explore the path from technical feasibility to clinical application. Moreover, this clearly demonstrates the timeliness of such reviews. By documenting both known and unknown information and emphasizing the importance of methodological rigor, we provide a well‐founded starting point for researchers entering this field.

## Author Contributions

All authors contributed to the article and approved the submitted version.

## Conflicts of Interest

The authors declare no conflicts of interest.

## Data Availability

The authors have nothing to report.
